# Interaction between Nuclear Receptor and Alpha-Adrenergic Agonist Subtypes in Metabolism and Systemic Hemodynamics of Spontaneously Hypertensive Rats

**DOI:** 10.1155/2024/5868010

**Published:** 2024-06-07

**Authors:** Sheryar Afzal, Munavvar Abdul Sattar, Ibrahim Albokhadaim, Ali Attiq, Mahmoud Kandeel, Aimi Syamima Abdul Manap, Sameer M. Alhojaily

**Affiliations:** ^1^ Department of Biomedical Science College of Veterinary Medicine King Faisal University, Al Hofuf, Saudi Arabia; ^2^ Discipline of Pharmacology School of Pharmaceutical Sciences Universiti Sains Malaysia, Gelugor 11800, Penang, Malaysia

## Abstract

Partial and full PPAR-*γ* agonists have shown promising effects and antihypertensive and antidiabetic agents through increased plasma adiponectin concentration. This study is aimed at examining the role of PPAR-*γ*, alpha-adrenoceptors, and adiponectin receptors in the modulation of vasopressor responses to angiotensin II (Ang II) and adrenergic agonists, after a subset treatment of partial and full PPAR-*γ* agonists, each individually, and also when coupled with adiponectin in SHRs. The antioxidant potential and metabolic indices for these animals were also determined. Group I (WKY) and group II (SHR) were designated as normotensive control and hypertensive control, respectively. Groups III (SHR) and IV (SHR) received irbesartan (30 mg/kg) and pioglitazone (10 mg/kg) orally for 28 days, and groups V (SHR), VI (SHR), and VII (SHR) were treated with adiponectin (2.5 *μ*g/kg) intraperitoneally alone, in combination with irbesartan, and in combination with pioglitazone, respectively, from days 21 to 28 only. On day 29, sodium pentobarbitone (60 mg/kg) was used to anesthetize all test animals, and systemic hemodynamic and plasma adiponectin concentrations and *in vitro* and *in vivo* antioxidant potential were measured. As compared to the WKY control, the SHR control group's noninvasive blood pressure and basal mean arterial pressure were significantly greater, along with increased arterial stiffness, lower plasma nitric oxide, adiponectin concentration, and antioxidant enzyme levels (all *P* < 0.05). However, they were gradually normalized by single drug treatments in all groups, and to a greater extent in the SHR + Irb + Adp group (*P* < 0.05). In the acute study, the dose dependant mean arterial pressure responses to intravenously administered adrenergic agonists and angiotensin-II were significantly larger in SHRs as compared to WKY by 20-25%. Adiponectin alone and in combination significantly blunted vasopressor responses to these alpha-adrenergic agonists in the SHR + Pio + Adp group by 63%, whereas attenuated responses to ANG-II administration to 70% in SHR + Irb + Adp. In conclusion, the combined treatment of adiponectin with PPAR-agonists reduced the systemic vascular responses to adrenergic agonists and improved arterial stiffness. This an evidence of the interaction of adiponectin receptors, PPAR-*γ*, alpha-adrenoceptors, and ANG-II in the systemic vasculature of SHRs. A significant level of synergism has also been proved among full PPAR-*γ* agonists and adiponectin receptors.

## 1. Introduction

Members of the receptor family peroxisome proliferator-activated receptors (PPARs) are highly concentrated in adipose tissue [1], vascular smooth and endothelial cells, renal glomerular cells [2], and skeletal muscle [3]. Insulin sensitivity is controlled in part by PPAR-*γ*, which is overexpressed in fat tissues [4]. The thiazolidinedione pioglitazone and other ligands boost the transcriptional regulation of genes involved in glucose and lipid metabolism [5, 6], leading to an increase in the number of small adipocytes [7]. The adipocyte phenotype and fat distribution can be altered by activating it with the thiazolidinedione (TZD) derivative pioglitazone [8], which triggers higher expression of fatty acid metabolism and triglyceride accumulation genes [9, 10].

Particularly successful in treating hypertension and heart failure [11] is the selective angiotensin-II AT1 receptor blocker irbesartan. Evidence from the scientific literature indicates that angiotensin receptor blockers (ARBs) induce adiponectin expression by acting as a partial PPAR-*γ* agonist by increasing PPAR-*γ* expression [12]. In addition, RAAS blockers, such as irbesartan, are shown to raise plasma adiponectin levels more than other antihypertensive drugs, including ACE inhibitors, AT1-antagonists, and calcium channel blockers [13]. These antihypertensive medications are helpful because they reduce oxidative stress [14], one of their many mechanisms of action. This means that ARB medication not only reduces blood pressure [15] but also reduces pulse wave velocity (PWV) [16], which in turn reduces arterial stiffness [15].

When the sympathetic nervous system (SNS) is overactive in response to physiological stress events, it causes an increase in heart rate and peripheral vascular resistance, both of which contribute to hypertension [17]. Our previous laboratory work with spontaneously hypertensive rats (SHRs) [18, 19] demonstrates an interaction between Ang-II and *α* 1adrenergic receptors in the vasculature of these animals. Adiponectin has been detected in cerebrospinal fluid (CSF), and its administration has a significant impact on energy homeostasis [20], suggesting that it may play a role in the control of SNS activity in the brain. It has also been shown that hypertension is linked to lower plasma adiponectin levels [21]. Adiponectin also plays a role in reducing oxidative stress, which helps prevent atherosclerosis ([22]. Because of leptin's central role in SNS activation and blood pressure regulation, it is intriguing to consider the possibility that adiponectin may regulate SNS activity by inhibiting leptin's brain-based functions. [23]. Additionally, adiponectin levels in the blood were lowered after angiotensin-II (Ang-II) infusions were administered chronically at modest dosages to control blood pressure [24]. The precise linkages and mechanisms linking decreased adiponectin production and low levels of nitric oxide (NO) to the development of hypertension remain unclear. Patients with hypertension and vascular diseases may benefit from PPAR-*γ* ligand intervention. However, whether or not the PPAR-*γ*, ATI, and *α* 1-adrenergic receptors in the vascular smooth muscle work together to alter vasomotor function in the hereditary model of hypertensive rats (SHRs) is unknown. Based on this information, we postulate that the various subtypes of alpha-adrenoceptors in SHRS work together with PPAR-*γ* to control blood pressure. Therefore, the primary objective of this study was to determine if the pharmacodynamics of vasopressor responses to intravenous administration of alpha-adrenergic agonists and Ang-II in SHRs would be affected by pretreatment with PPAR-*γ* agonists alone or in combination with exogenously administered adiponectin. Adiponectin and these PPAR-*γ* agonists were also tested *in vivo* and *in vitro* for their synergistic influence on arterial stiffness fluctuations and the systemic vasculature's response to intravenously administered subtypes of alpha-adrenergic agonists and Ang-II.

## 2. Materials and Methods

### 2.1. Experimental Animals and Treatments

A total of 42 spontaneously hypertensive rats (SHR) obtained from the Animal Research Unit and Service Centre of Universiti Sains Malaysia, weighing an average of 230-255 g, were randomly assigned to seven groups and kept in stainless metabolic cages for three days to adapt before beginning medication pretreatment (*n* = 6/group); a control group of six WKY rats also participated. All rats were given free access to commercial rat chow (Gold Coin Sdn. Bhd., Penang, Malaysia) and running water. The Animal Ethics Committee at the University Sains approved all protocols and procedures involving animal care and treatment.

Malaysia (AECUSM) with approval reference no.: USM/Animal Ethics Approval/2012/(75) (352).

The animals were randomly categorized into 7 groups (*n* = 6) and were named as follows:

*Group I:* WKY control (WKY + VEH) served as normotensive control and treated with vehicle.

*Group II:* SHR control (SHR+ VEH) served as SHR control and treated with vehicle.

*Group III:* treated with irbesartan (SHR + Irb) @ 30 mg/kg orally from day 1 to day 28.

*Group IV:* treated with pioglitazone (SHR + Pio) @ 10 mg/kg orally from day 1 to day 28.

*Group V:* treated with adiponectin (SHR + Adp) @ 2.5 *μ*g/kg intraperitoneally from days 21 to 28 only.

*Group VI:* treated with a combination of irbesartan (SHR + Irb + Adp) @ 30 mg/kg orally from day 1 to day 28 and adiponectin @ 2.5 *μ*g/kg intraperitoneally from days 21 to 28.

*Group VII:* treated with a combination of pioglitazone (SHR + Pio + Adp) @ 10 mg/kg orally from day 1 to day 28 and adiponectin @ 2.5 *μ*g/kg intraperitoneally from days 21 to 28.

Each drug's stock solution was renewed every day. Stock solutions of 30 mg/ml and 10 mg/ml of irbesartan (Aprovel, Sanofi, Aventis, France) and pioglitazone (Searle, Pvt., Ltd., Pakistan) were prepared by dissolving tablets of each drug in distilled water. Two hundred milliliters of phosphate-buffered saline (PBS) were infused with 2.5 milligrams per kilogram (25 mg/kg) of full-length recombinant adiponectin (Chemtron Biotechnology Sdn. Bhd.) [25].

### 2.2. Alpha-Adrenergic Agonists

Noradrenaline is a mixed adrenoceptor agonist, which acts on *α*1 and *α*2-adrenoceptors, whereas phenylephrine (PE) reacts only on *α*1-adrenoceptors to stimulate adrenoceptors subtypes including *α* 1A-, *α* 1B-, and *α* 1D, noradrenaline (Na) works on both *α* 1 and *α* 2adrenoceptors. In addition, methoxamine (ME) is selective for alpha 1A adrenoceptors solely [26]. Angiotensin II (Ang II), when bound to AT1 receptors, causes systemic vasoconstriction [19], while the agonists employed in this work were freshly produced in saline.

### 2.3. Systemic Vasopressor Response Study

Using a single-phase acute vasopressor responses experiment, we compared the sensitivity of the systemic vasculature of control WKY, SHR, and pretreated SHRs to exogenously administered Ang-II and adrenergic agonists and assessed the functional role of *α* 1adrenoceptor subtypes in the systemic vasculature. The peripheral vasculature was demonstrated to respond to vasopressors by constricting, leading to an increase in blood pressure after their administration. To achieve this, ascending and descending bolus doses of noradrenaline (200, 400, and 800 ng), phenylephrine (2, 4, and 8 *μ*g), methoxamine (2, 4, and 8 *μ*g), and angiotensin II (5, 10, and 20 ng) were injected intravenously into the left jugular vein. Each agonist was given a 10-minute break in between uses to ensure they were thoroughly washed away. While resting MAP was continuously monitored throughout the experiment, it was only the MAP readings immediately after agonist infusion that were used to determine the basal values.

### 2.4. Physiological Data Collection (Basal and Follow-Up Metabolic and Antioxidant Parameters)

On day 0 of the trial, the baseline variables were determined by measuring blood glucose, body weight, a 24-hour urine collection, and water intake. We repeated these measurements on days 8, 21, and 28 of the experiment. Daily urine collections were accompanied by a blood draw, with the plasma separated after centrifugation at 3500 rpm for 10 minutes. Biochemical analysis of the plasma and urine samples necessitated freezing them at -30 degrees Celsius. Following the instructions provided by the Institute of Biological Engineering at Nanjing Jianchen, Nanjing, China, spectrophotometric detection kits were to determine the concentrations of nitric oxide (NO) and total antioxidative activity (TAOC) in the plasma samples of SHRs that had been collected. The adiponectin levels in the blood were estimated using a Chemtron, Biotechnology Sdn, Malaysian max rat adiponectin ELISA kit.

### 2.5. Measurement of Conscious or Noninvasive Blood Pressure (NIBP)

The gold standard for a precise assessment of blood pressure is a direct blood pressure measurement performed through an invasive surgical procedure. However, researchers have sought to measure blood pressure noninvasively for the past 35 years with varied degrees of success. It has been suggested that when comparing invasive blood pressure versus noninvasive blood pressure, direct blood pressure should be measured on a rodent's carotid artery (Bunag, 1973). The CODA noninvasive blood pressure device (Kent Scientific Corporation, Torrington, Connecticut, USA) was used to measure the noninvasive blood pressure (NIBP). The CODA data acquisition system is linked to Hewlett Packard Centrino core I3 computer with Windows 7 operating system and measurement of NIBP was done using data acquisition software version 4.1 (Kent Scientific Corporation). There were three sets of measurements, each consisting of ten cycles. The first 10 rounds were set aside as “acclimatization” rounds. When assessing blood pressure, the mean of the last 20 cycles was utilized as a benchmark. Following a 3-day acclimation period, the SHR rats underwent NIBP measurements (tail-cuff method) for another 3 days in a row to determine their resting blood pressures. Only SHRs with systolic blood pressures greater than 150 mm Hg were used in this study.

### 2.6. Systemic Hemodynamics Data and Measurement of Arterial Stiffness

Every animal underwent a 12- to 14-hour fast before the surgical procedures were done for the hemodynamic evaluation. All cannulae and the transducer were refilled with heparinized saline (20.0 units/ml). To begin, 60 mg/kg sodium pentobarbitone (Nembutal, CEVA Sante Animale, Libourne, France) was injected intraperitoneally into each animal to induce anesthesia. The airway was cleared by inserting a PP240 tube into the trachea, and the animal was hastily hooked up to a ventilator (Small animal ventilator, Harvard Apparatus, Edinburgh, UK) to help it breathe. After isolating and catheterizing the left jugular vein with a PP50 cannula, a 50 ml syringe attached to an infusion pump (Perfusor Secura FT 50 ml, B. Braun) was inserted into the vein to infuse normal saline at a flow rate of 3 ml/hr. The left carotid artery was then catheterized with a PP50 (internal diameter 0.58 mm) tube so systemic arterial blood pressure could be measured directly using a pressure transducer (P23 ID Gould, Statham Instruments Inc., USA) connected to a computerized data acquisition system (Powerlab, ADInstruments Australia).

**Figure 1:**
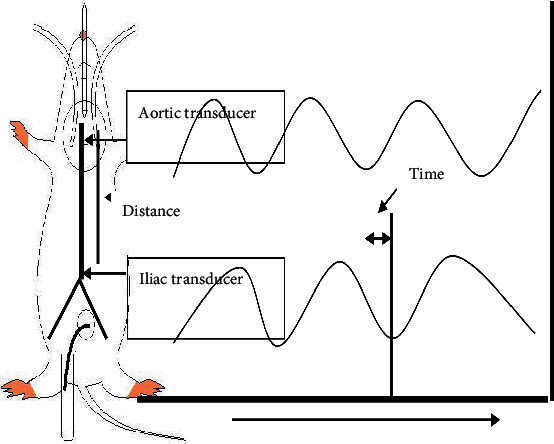
Presentation of propagation time and distance for measurement of PWV.

A midline abdominal incision was made to expose the left kidney. To finish the dissection, an electrical cautery knife was employed (Rimmer Brothers, UK). Following standard laboratory protocol, a PP50 catheter (internal diameter 0.58 mm) was used to catheterize the left iliac artery and measure arterial stiffness in terms of pulse wave velocity, defined as the product of the propagation distance (*d*) and the propagation time (*t*) in meters per second. Swarup et al. [27]. The propagation time was estimated using a manual “foot to foot” method [28]. The delay between the upstrokes (foot) of each pressure wavefront was used to manually determine the time required for the pulse wave (*t*) to travel from the aortic arch to the abdominal aorta. Manual foot-to-foot measurement is widely used since it is considered an accurate method of determining PWV. When calculating the time it takes for a pulse wave to travel from the aortic arch to the abdominal aorta, the delay between the upstroke (foot) of each pressure wavefront was taken into account. The duration of ten consecutive regular heartbeats was utilized to calculate the propagation time. A method for calculating the distance and time required for a signal to travel is depicted schematically in Figure 1. At the end of the experimental recordings, the animal was euthanized with an overdose of sodium pentobarbitone (200 mg/kg), and the full length of the aorta was exposed to measure the PWV. A damp silk thread was placed along the contour of the aorta and marked at the tips of the two cannulae. The thread was then removed and laid straight, and the distance between the two marks was measured. This pulse wave propagation distance was used to calculate the PWV. Any data from a cycle in which an abnormal waveform was discovered was destroyed and replaced with data from the following cycle in which a normal waveform was observed

### 2.7. Data Analysis

Metabolic data such as body weight, fluid consumption, blood glucose level, plasma adiponectin concentration, and systemic haemodynamic parameters such as systolic blood pressure, diastolic blood pressure, mean arterial pressure, and heart rate were analyzed using repeated-measures one-way ANOVA during the experimental treatment period. One-way analysis of variance was performed on plasma nitric oxide, total antioxidant capacity, and adiponectin concentrations. Vasopressor responses (increase in MAP) induced by Ang-II and adrenergic agonists were also averaged across all doses of each agonist administered in ascending and descending order. Two-way ANOVA was used to compare the dosage differences, and one-way ANOVA was used to compare the means. The complete data presented in this study were expressed as Mean ± S.E.M, and differences between the means were considered significant at a 5% level (*P* < 0.05), as the standardized analytical significance value for all parameters observed in this study; comparison was made between treated and the respective control groups. Additionally, the statistical analysis for this part was performed using GraphPad Prism® software version 5.00 for Windows (GraphPad Software, San Diego, CA).

## 3. Results

### 3.1. Metabolic Indices and Plasma Analysis

According to the study protocol, observations for changes in metabolic parameters including body weight, water intake, and urine flow of all seven groups of rats were made on four different occasions (day 0, day 8, day 21, and day 28) of the study and presented in Table 1. At the onset of the treatment period, the basal body weights in each group were equivalent, and the vehicle-treated WKY and SHR groups did not vary from each other in their age-dependent body weight gains throughout the treatment (*P* > 0.05). The WKY and SHR control groups were not significantly different from one another on any of the four observation days (*P* > 0.05). The age-dependent increase in body weight was observed in irbesartan and pioglitazone-treated groups. The body weight significantly increased at days 21 and 28 in comparison to their initial basal body weight at day 0, respectively (*P* < 0.05) (Table 1). However, the final body weight was not significantly different between all groups on day 28, except the groups treated with adiponectin alone or in combination with either irbesartan or pioglitazone for one week as compared to their counterpart control SHR+ VEH group from day 21 to day 28, respectively (*P* < 0.05) (Table 1). Conclusively, the body weight was significantly decreased in adiponectin-treated groups only, whereas irbesartan and pioglitazone treatment had no significant difference as compared to the SHR+ VEH group as the study progressed.

The mean values of daily water intake and urine flow rate for each of the seven groups of rats were observed on the same pattern of observational days during the study period and are presented in Table 1. On all four observation days, it was noted that the SHR + CON group's water intake was significantly less than that of the WKY + VEH group (*P* < 0.05) (Table 1). No significant difference in water intake was observed in the SHR + VEH group as the mean 24 hr water intake remained constant for SHR+ VEH, SHR + Irb, and SHR + Pio groups throughout the study period (*P* > 0.05) (Table 1). By contrast, one-week adiponectin treatment significantly decreased water intake in SHR + Adp, SHR + Irb + Adp, and SHR + Pio + Adp treated groups from days 21 to 28 in comparison to SHR + VEH group (*P* < 0.05) (Table 1).

It was also evident that the urine flow rate was significantly lower in the SHR + VEH in comparison to the WKY + CON group on all 4 observational days WKY + VEH vs. SHR + VEH group (*P* < 0.05) (Table 1). The separate treatment of irbesartan and pioglitazone did not pose any significant effect on the urine flow rate of SHR-treated groups on the observational days. However, treatment of adiponectin from day 21 to day 28 alone and in combination with either irbesartan or pioglitazone increased the urine flow rate of SHR + Adp, SHR + Irb + Adp, and SHR + Pio + Adp groups significantly on day 28 only (*P* < 0.05) (Table 1). Interestingly, the SHR + Irb + Adp and SHR + Pio + Adp groups showed an increased urine flow rate as compared to SHR + Adp on day 28 (*P* < 0.05) (Table 1).

**Figure 2:**
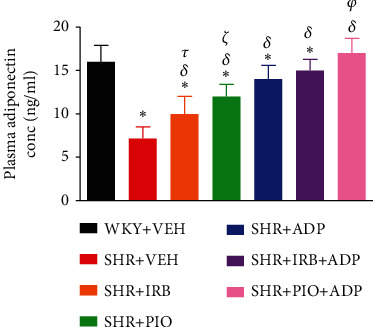
Plasma adiponectin concentration of experimental control and treated groups. The values are presented as mean ± S.E.M. (*n* = 6) in each group and were analyzed by one-way. ANOVA followed by the Bonferroni *post hoc* test. Values with *P* < 0.05 were considered statistically significant. ^∗^*P* < 0.05 versus WKY + VEH. ^*δ*^*P* < 0.05 versus SHR + VEH. ^*τ*^Significant difference between SHR + Irb and SHR + Adp. ^*ξ*^Significant difference between SHR + Pio and SHR + Adp. ^*φ*^Significant difference between SHR + Adp and SHR + Pio + Adp.

The mean values of plasma adiponectin concentration of experimental groups were measured on day 28 only of the study and are shown in Figure 2. A significant decrease in plasma adiponectin concentration was noted in SHR + CON in comparison to the WKY + VEH group (7.2.0 ± 1.3 vs. 16 ± 1.9 *μ*g/ml), (*P* < 0.05). Treatment with irbesartan (30 mg/kg/day), pioglitazone (10 mg/kg/day), and adiponectin (2.5 *μ*g/kg/day) significantly rise the plasma adiponectin levels in SHR + Irb, SHR + Pio, and SHR + Adp groups in comparison to SHR + VEH group (i.e., 10.1 ± 1.1, 12.20 ± 1.4, and 14.78 ± 1.7 vs. 7.2 ± 1.3 ng/ml), respectively, (*P* < 0.05). The concurrent treatment of adiponectin with SHR + Irb + Adp and SHR + Pio + Adp dramatically raised adiponectin levels in the plasma, whereas the degree of increment in the SHR + Pio + Adp group was significantly larger (*P* < 0.05) in comparison to SHR + Irb + Adp. Surprisingly the values obtained were comparable to the WKY + VEH group (i.e., 17.230 + 1.7 vs. 16.14 + 1.3 ng/ml), respectively (*P* > 0.05) (Figure 2).

### 3.2. Plasma Nitric Oxide and Total Antioxidant Capacity

**Figure 3:**
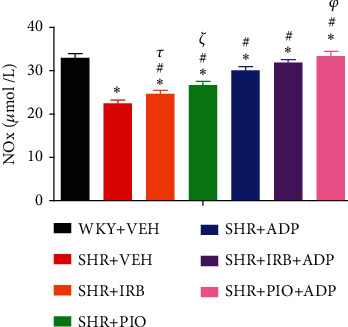
Plasma nitric oxide levels of experimental control and treated groups. The values are presented as mean ± S.E.M.(*n* = 6) in each group and were analyzed by one-way ANOVA followed by the Bonferroni *post hoc* test. Values with *P* < 0.05 were considered statistically significant. ^∗^*P* < 0.05 versus WKY + VEH. ^#^*P* < 0.05 versus SHR + VEH. ^*τ*^Significant difference between SHR + Irb and SHR + Adp. ^*ξ*^Significant difference between SHR+ Pio and SHR + Adp. ^*φ*^Significant difference between SHR + Adp and SHR + Pio + Adp groups.

Plasma nitric oxide (NO) levels of the SHR control group were significantly lower in comparison to the WKY + VEH group (i.e., 22.54 ± 0.77 vs. 33.12 ± 0.99 nmol/mL), respectively (*P* < 0.05) (Figure 3). When compared to the SHR + CNT group, the pioglitazone and adiponectin treatment significantly augmented plasma NO levels in the SHR + Irb and SHR + Pio groups (i.e., 24.75 ± 0.81 and 26.79 ± 0.89 vs. 22.54 ± 0.77 nmol/mL), respectively (*P* < 0.05) (Figure 3). Moreover, the SHR + Adp group demonstrated greater plasma NO level than the SHR + Irb and SHR + Pio groups (30.17 ± 0.74) *μ*mol/L. The plasma NO level was significantly raised when adiponectin and pioglitazone were administered together in SHR + Pio + Adp as compared to SHR + Irb + Adp-treated group (i.e., 31.98 ± 1.01 vs. 33.52 ± 0.64 *μ*mol/L) (*P* < 0.05) (Figure 3).

**Figure 4:**
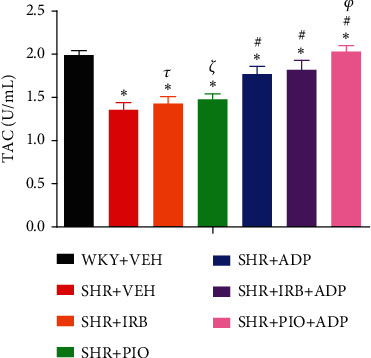
Plasma total antioxidant capacity of experimental control and treated groups. The values are presented as mean ± S.E.M. (*n* = 6) in each group and were analyzed by one-way ANOVA followed by the Bonferroni *post hoc* test. Values with *P* < 0.05 were considered statistically significant. ^∗^*P* < 0.05 versus WKY + VEH. ^#^*P* < 0.05 versus SHR + VEH. ^*τ*^Significant difference between SHR + Irb and SHR + Adp. ^*ξ*^Significant difference between SHR+ Pio and SHR + Adp. ^*φ*^Significant difference between SHR + Adp and SHR + Pio + Adp groups.

Similarly, data for mean values of total antioxidant capacity (TAOC) of blood plasma and plasma nitric oxide of all the seven experimental groups of rats are presented in Figure 4. When compared to WKY control, it was found that the TAOC of the blood plasma level in the SHR control groups of rats was significantly reduced (i.e., 1.37 ± 0.09 vs. 1.99 ± 0.09 U/mL), respectively (*P* < 0.05) (Figure 4). The independent administration of pioglitazone or irbesartan did not pose any significant effect in TAOC in SHR + Irb, SHR + Pio as compared to the SHR + VEH group (i.e., 1.43 ± 0.10 and 1.47 ± 0.09 vs. 1.36 ± 0.09 U/mL), respectively. The adiponectin-treated group alone and in combination with the pioglitazone group significantly increased the TAOC of the blood plasma sample (i.e., 1.65 ± 0.11 and 1.97 ± 0.06 U/mL), respectively (*P* < 0.05). There was no significant difference between SHR + Adp and SHR + Irb + Adp groups for plasma TAOC values (*P* > 0.05) (Figure 4).

### 3.3. Systemic Haemodynamics Blood Pressure Measurements

The changes in mean values of blood pressure responses (SBP, DBP, MAP, and HR) of all the seven experimental groups of rats were measured by using the tail-cuff method and were obtained in a conscious state on four days of observation (days 0, 8, 21, and 28) during the study period. The mean values of systolic blood pressure (SBP) responses of all seven groups of rats are shown in Table 2. On each of the four observational days, SBP in the SHR + VEH group was found to be significantly greater than in WKY + VEH (*P* < 0.05) (Table 2). Similarly, SBP of SHR + Irb, SHR + Pio, SHR + Adp, SHR + Irb + Adp, and SHR + Pio + Adp groups remained significantly higher on days 0, 8, and 21 of observation as compared to normotensive WKY control (*P* < 0.05). The SHR + Irb and SHR + Pio groups demonstrated decreased SBP on day 21 in comparison to the SHR + VEH group (*P* < 0.05), whereas on day 28, treatment with adiponectin either alone or in combination with irbesartan or pioglitazone further decreased SBP in SHR + Adp, SHR + Irb + Adp, and SHR + Pio + Adp as compared to the SHR + VEH group (*P* < 0.05) (Table 2). However, the tendency to decrease SBP in the SHR + Irb + Adp group was significantly greater in contrast to SHR + Pio + Adp on day 28 only, and the outcomes attained therein were equivalent to those of the WKY + VEH group (all *P* < 0.05) (Table 2).

Similarly, the mean values of diastolic blood pressure (DBP) of all experimental groups are presented in Table 2. It was noticed that DBP was significantly greater in SHR + VEH in contrast to WKY + VEH on all four observational days (*P* < 0.05) (Table 2). It was found that the DBP of the experimental rat groups SHR, SHR + Pio, SHR + Irb, SHR + Adp, and SHR + Pio + Adp remained significantly (*P* < 0.05) elevated on all four points of observation in contrast to the WKY + VEH group. The SHR + Irb, SHR + Pio, SHR + Adp, SHR + Pio + Adp, and SHR + Irb + Adp groups exhibited decreased DBP on day 28 only as compared to the SHR + VEH group (*P* < 0.05) (Table 2). However, combined treatment with adiponectin in SHR + Irb + Adp and SHR + Pio + Adp significantly decreased DBP on day 28 as compared to their separate treatment and was of larger extent in SHR + Irb + Adp in contrast to other sets of combined treatment (all *P* < 0.05) and was comparable to values attained in the WKY + VEH group (Table 2).

The values of mean arterial pressure (MAP) followed the same pattern of significant changes in the observational days as that of SBP and DBP values for control and treated groups and are presented in Table 2. Here again, the MAP was significantly greater in SHR + CON in comparison to WKY + COT (*P* < 0.05), whereas the SHR + Irb group demonstrated reduced MAP on day 21 in comparison to the SHR + CON group. Moreover, on day 28, adiponectin therapy further lowered MAP values both on its own and when combined with either irbesartan or pioglitazone in SHR + Adp, SHR + Irb + Adp, and SHR + Pio + Adp groups as compared to SHR + VEH group (*P* < 0.05). The tendency to decrease the values in SHR + Irb + Adp group was significantly lower as compared to SHR + Pio + Adp with a comparative trend for the values obtained in WKY + VEH (*P* < 0.05) (Table 2). Throughout the course of the study, the experimental rat SHR groups' heart rates (HR) stayed substantially higher than those of WKY + VEH, i.e., days 0, 8, 21, and 28. No significant difference was found on days 0, 8, and 21 in SHR-treated groups in comparison to the SHR control group. Single treatment with pioglitazone, irbesartan, and adiponectin and in combination with adiponectin on day 28 only significantly decreased HR in SHR + Irb, SHR + Pio, SHR + Adp, SHR + Irb + Adp, and SHR + Pio + Adp in comparison to the SHR + CON group (*P* < 0.05). However, none of the values was comparable to those of the WKY + CON group (*P* < 0.05) (Table 2).

### 3.4. Pulse Wave Velocity

**Figure 5:**
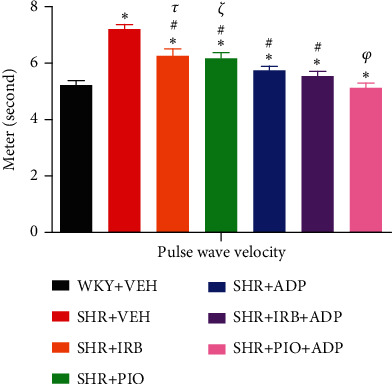
Pulse wave velocity of experimental control and treated groups. The values are presented as mean ± S.E.M. (*n* = 6) in each group and were analyzed by one-way ANOVA followed by the Bonferroni *post hoc* test. Values with *P* < 0.05 were considered statistically significant. ^∗^*P* < 0.05 versus WKY + VEH. ^#^*P* < 0.05 versus SHR + VEH. ^*τ*^Significant difference between SHR + Irb and SHR + Adp. ^*ξ*^Significant difference between SHR + Pio and SHR + Adp. ^*φ*^Significant difference between SHR + Adp and SHR + Pio + Adp groups.

Mean values of pulse wave velocity (PWV) of all the seven experimental groups of rats under observation are given in supplementary data.  Figure 5 depicts the changes in PWV in the SHR + CON vs. WKY + VEH group, i.e., 7.21 ± 0.16 vs. 5.23 ± 0.15 rn/s. Irbesartan 30 mg/kg/day (6.26 ± 0.25) m/s, 10 mg/kg/day of pioglitazone (6.18 ± 0.17) m/s, and 2.5 *μ*g/kg/day of adiponectin (5.69 ± 0.13) m/s blunted the increase in PWV in SHR-treated groups. Nevertheless, compared to groups treated with either irbesartan or pioglitazone singly, the tendency to decrease PWV in the adiponectin-treated groups was significantly greater (*P* < 0.05). However, compared to the SHR + Irb + Adp group, which did not differ from the SHR + Adp group, the combination therapy of adiponectin and pioglitazone in the SHR + Pio + Adp group (5.130.27) further decreased the PWV and reached the level of WKY + VEH group (5.230.23 m/s) (*P* > 0.05) (Figure 5).

### 3.5. Systemic Vasopressor Responses Study

It was found that both individual and combined treatments of adiponectin with either irbesartan or pioglitazone affected vasopressor responses to Ang-II and adrenergic agonists in SHR. The systemic vasopressor response experiment involved the administration of bolus doses of Ang-II and adrenergic agonists such that each dose produced an increase in the magnitude of MAP due to the vasopressor action of these agonists. The increase in MAP due to these agonists was dose-dependent, whereas the adrenergic agonists utilized in the study were noradrenaline (NA), phenylephrine (PE), and methoxamine (ME).

### 3.6. Noradrenaline (NA)

**Figure 6:**
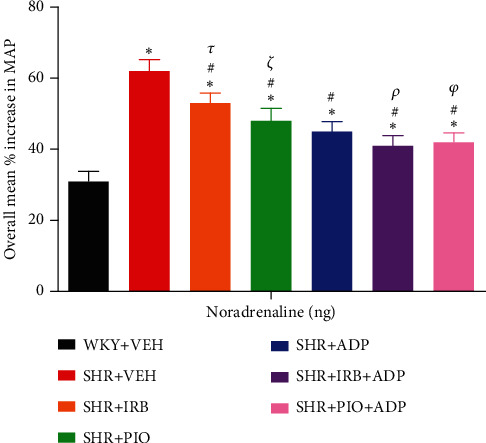
Overall % change increase in MAP in response to graded doses of noradrenaline in experimental control and treated groups. The values are presented as mean ± S.E.M. with (*n* = 6) in each group and were analyzed by one-way ANOVA followed by the Bonferroni *post hoc* test. Values with *P* < 0.05 were considered statistically significant. ^∗^*P* < 0.05 versus WKY + VEH. ^#^*P* < 0.05 versus SHR + VEH. ^*τ*^Significant difference between SHR + Irb and SHR + Adp. ^*ξ*^Significant difference between SHR + Pio and SHR + Adp. ^*φ*^Significant difference between SHR + Adp and SHR + Pio + Adp groups.

Upon infusion intravenously, exogenously administered NA resulted in dose-related increases in MAP in all experimental groups, whereas the overall percentage increase in MAP which is the mean of three responses to three doses of the agonist is presented in Table 3. In the SHR + VEH group, as compared to the WKY + VEH group, the entire spike in MAP in response to exogenously delivered NA was significantly larger (SHR + VEH vs. WKY + VEH: 62 ± 3 vs. 31 ± 2%) (*P* < 0.05). The magnitude of MAP increase due to intravenously administered NA was significantly blunted after treatment with irbesartan (SHR + Irb vs. SHR + VEH: 53 ± 2 vs. 62 ± 3%), pioglitazone (SHR + Pio vs. SHR + VEH: 48 ± 2 vs. 62 ± 3%), and adiponectin (SHR + Adp vs. SHR + VEH: 45 ± 3 vs. 62 ± 3%). The overall increase in MAP in response to NA treated with adiponectin and irbesartan and adiponectin with pioglitazone-treated rats was further significantly reduced in comparison to their separate treatment of adiponectin in SHR-treated rat groups (SHR + Irb + Adp and SHR + Pio + Adp vs. SHR + Adp: 40 ± 2 and 41 ± 1 vs. 45 ± 1%) (Figure 6, Table 3) (*P* < 0.05).

### 3.7. Phenylephrine (PE)

**Figure 7:**
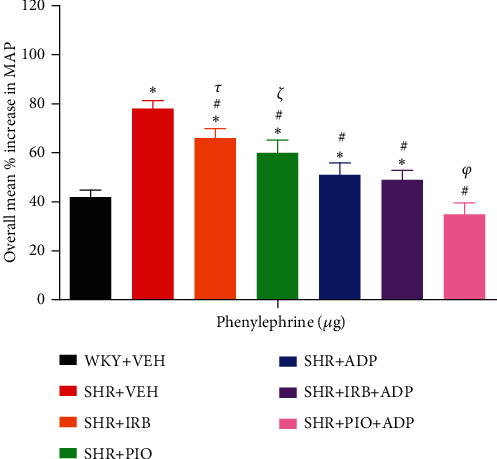
Overall % change increase in MAP in response to graded doses of phenylephrine in experimental control and treated groups. The values are presented as mean ± S.E.M. (*n* = 6) in each group and were analyzed by two-way ANOVA followed by the Bonferroni *post hoc* test. Values with *P* < 0.05 were considered statistically significant. ^∗^*P* < 0.05 versus WKY + VEH. ^#^*P* < 0.05 versus SHR + VEH. ^*τ*^Significant difference between SHR + Irb and SHR + Adp. ^*ξ*^Significant difference between SHR + Pio and SHR + Adp. ^*φ*^Significant difference between SHR + Adp and SHR + Pio + Adp groups.

Similar to NA, PE resulted in dose-dependent increases in MAP in all experimental groups (Figure 7). The overall percentage increase in MAP to three doses of the alpha-adrenergic agonist was significantly greater in the SHR + VEH rats in comparison to the WKY + VEH group (i.e., SHR + VEH vs. WKY + VEH: 78 ± 4 vs. 42 ± 3%) (*P* < 0.05). The magnitude of MAP increase due to PE administration was significantly blunted after irbesartan treatment (i.e., SHR + Irb vs. SHR + VEH: 66 ± 3 vs. 78 ± 4%), pioglitazone (SHR + Pio vs. SHR + VEH: 60 ± 3 vs. 78 ± 4%), and adiponectin (SHR + ADP vs. SHR + VEH: 51 ± 3 vs. 78 ± 4%). The overall increase in MAP in response to PE administration to groups treated with adiponectin with irbesartan and adiponectin with pioglitazone was further significantly reduced in comparison to separate treatment of adiponectin (i.e., SHR + Irb + Adp and SHR + Pio + Adp vs. SHR + Adp: 49 ± 3 and 35 ± 4 vs. 51 ± 23%) (Figure 7, Table 3) (*P* < 0.05). There was no discernible difference between the SHR + Pio + Adp group and the WKY + VEH group (SHR + Pio + Adp vs. WKY + VEH: 35 ± 4 vs. 42 ± 3%) (Figure 7, Table 3) (*P* < 0.05).

### 3.8. Methoxamine (ME)

**Figure 8:**
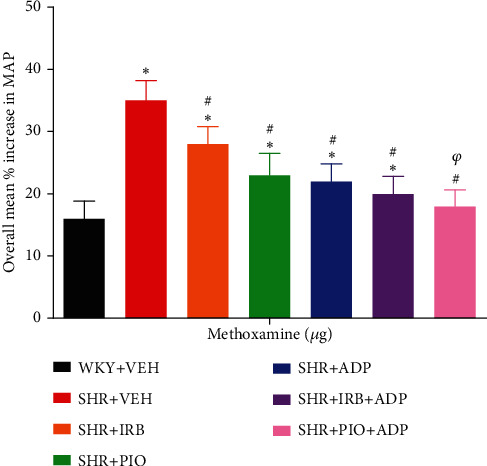
Overall % change increase in MAP in response to graded doses of methoxamine in experimental control and treated groups. The values are presented as mean ± S.E.M. (*n* = 6) in each group and were analyzed by two-way ANOVA followed by the Bonferroni *post hoc* test. Values with *P* < 0.05 were considered statistically significant. ^∗^*P* < 0.05 versus WKY + VEH. ^#^*P* < 0.05 versus SHR + VEH. ^*φ*^Significant difference between SHR + Adp and SHR + Pio + Adp groups.

Methoxamine was noted to produce vasoconstrictions in a dose-dependent pattern as a result of intravenous administration in all experimental groups (Figure 8), whereas the overall percentage increase in MAP was significantly higher in the SHR + VEH group in comparison to WKY + VEH (i.e., SHR + VEH vs. WKY + VEH: 35 ± 3 vs. 16 ± 2%) (*P* < 0.05). Additionally, the extent of MAP increase resulting from ME administration was significantly blunted after treatment with irbesartan (SHR + Irb vs. SHR+ VEH: 28 ± 2 vs. 35 ± 3%), pioglitazone (SHR + Pio vs. SHR + VEH: 23 ± 3 vs. 35 ± 3%), and adiponectin (SHR + ADP vs. SHR+ VEH: 22 ± 2 vs. 35 ± 3%). The overall increase in MAP in response to ME treated with adiponectin and pioglitazone-treated rats was further significantly reduced as compared to separate treatment of adiponectin (SHR + Pio + Adp vs. SHR + Adp: 18 ± 2 vs. 22 ± 2%) and was comparable to the WKY + VEH group (*P* < 0.05). There was no significant difference in SHR + Irb + Adp and SHR + Adp groups (Figure 8, Table 3 (*P* < 0.05)).

### 3.9. Angiotensin II (Ang II)

**Figure 9:**
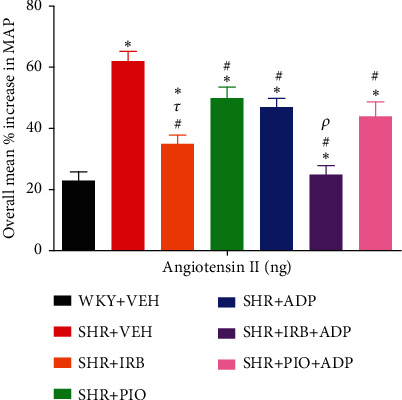
Overall % change increase in MAP in response to graded doses of angiotensin-II in experimental control and treated groups. The values are presented as mean ± S.E.M. (*n* = 6) in each group and were analyzed by two-way ANOVA followed by the Bonferroni *post hoc* test. Values with *P* < 0.05 were considered statistically significant. ^∗^*P* < 0.05 versus WKY + VEH. ^#^*P* < 0.05 versus SHR + VEH. ^*τ*^Significant difference between SHR + Irb and SHR + Adp. ^*ρ*^Significant difference between SHR+ Adp and SHR + Irb + Adp groups.

Ang-II exhibited an equivalent trend of results to NA, PE, and ME as it produced dose-dependent increases in MAP following pretreatments in all groups (Figure 9). The overall percentage increase in MAP was significantly higher in the SHR + CNT rats in comparison to the SHR + CNT group (i.e., SHR + VEH vs. WKY + VEH: 62 ± 3 vs. 23 ± 4%) (*P* < 0.05), but was significantly blunted following treatment with irbesartan as compared to the WKY + VEH group (SHR + Irb: 35 ± 4 vs. SHR + VEH: 62 ± 3), and was further reduced in combined treatment with adiponectin in SHR + Irb + Adp (25 ± 2) (*P* < 0.05) and was comparable to WKY + VEH group values. However, adiponectin treatment for 7 days also significantly decreased the overall percentage increase in MAP in SHR + Adp and SHR + Pio + Adp as compared to the SHR + VEH group (SHR + Adp vs. SHR + VEH: 47 ± 3 vs. 62 ± 3; SHR + Pio + Adp vs. SHR + VEH: 50 ± 4 vs. 62 ± 3%) (*P* < 0.05). No significant difference was noted in pioglitazone and the combination of adiponectin with pioglitazone in SHR + Adp and SHR + Pio + Adp groups (Figure 9, Table 3 (*P* < 0.05)).

## 4. Discussion

Accordingly, the present study is distinctive in the sense that it investigates and defines the impact of exogenously administered adiponectin on the classification and the interaction link between PPAR-*γ*, RAAS, and SNS, which could be involved in blood pressure regulation, and responses of the systemic vascular system to a subset of alpha-1-adrenoceptors (*α*1A, *α*1B, and *α*1D) and Ang-II modifications. Moreover, pharmacodynamically, *in vivo* and *in vitro*, the synergistic antioxidant potential of circulating levels of adiponectin and the integrity of PPAR-*γ* and adiponectin receptors (adipo R1 and adipo R2) in the presence of alpha-adrenergic agonists in SHRs were determined. The animals used in this study were the Okamoto strain of spontaneously hypertensive rats (SHR), which mimics a model of primary hypertension in humans and are widely used because of their similarity with human main cardiovascular characteristics. Since the 1960s, they have been employed to study the CVD diseases [29], and their kidney also performs a crucial part in the initiation and maintenance of high blood pressure [30]. Previous reports have proven that an interactive relationship exists between AT1 and *α*1-adrenoceptors in modulating adrenergically and Ang-II-induced vasoconstriction in the rat systemic and renal vasculature [18, 19]. Moreover, PPAR-*γ* interacts or a cross-talk relationship exists between PPAR-*γ*, alpha-adrenoceptor subtypes (*α*1A-, *α*1B-, and *α*1D), and Ang II receptors in the systemic vasculature of diabetic experimental rats [31]. However, there is a paucity of data regarding the effect of adiponectin administration on the functional contribution of *α*1-adrenoceptors subtypes in the systemic vasculature of diabetic hypertensive rats. Therefore, a set of adrenergic agonists and Ang-II have been used in the pretreated rats to underline the significance of their interaction.

### 4.1. Metabolic Indices

We observed a significant decrease in body weight in adiponectin-treated groups only either alone or in combination with PPAR-*γ* agonists. Previous study reported that adiponectin therapy reduces body weight because it stimulates fatty acid oxidation and glucose uptake by activating AMP-activated protein kinase [31]. Nevertheless, without affecting food intake [32], it eventually causes a reduction in adipose tissue mass by activating its receptors (adipoR1 and R2), which are mainly expressed in the arcuate and lateral hypothalamic nuclei [33]. Additionally, treatment with adiponectin alone or in conjunction with PPAR-*γ* agonists caused a drop in fluid intake as compared to their control counterparts, whereas in another study, an age-dependent rise in fluid consumption was induced by irbesartan administration in SHRs after Ang-II AT1R blockade [34]. Moreover, adiponectin-treated groups expressed diuresis in SHRs, which may be due to the way it inhibits the release of antidiuretic hormone and has natriuretic effects. This likely caused sodium ions to passively follow water in SHRs.

As hypoadiponectinemia is inversely proportional to hypertension [35], therefore, SHRs used in this study showed lower values of adiponectin as compared to normotensive rats. However, in comparison to other treatment patterns, we discovered that SHRs treated with adiponectin for 7 days in combination with full pioglitazone for 3 weeks had a significant rise in the levels of adiponectin. Noteworthy, adiponectin administration was at an extent sufficient to elevate its plasma concentration, which in turn decreased B. P and improved systemic vascular hemodynamics and were found to be similar to those achieved with PPAR-*γ* agonists. Therefore, plasma levels of adiponectin are increased by activating PPAR-*γ* with partial and full agonists, likely through promoting the expression of proteins necessary for adiponectin synthesis (endoplasmic reticulum oxidoreductin-1 protein) and release (disulfide-bond A oxidoreductase-like protein), [31], whereas we did not quantify these proteins in this study. Furthermore, pioglitazone, as a full PPAR-*γ* agonist, also attenuated B. P in SHRs directly via stimulating plasma adiponectin concentration. These observations regarding plasma adiponectin concentration run similar to our previous laboratory findings in various models of diabetic hypertensive [31, 36] and normotensive rats [37].

### 4.2. Blood Pressure Regulation

Adiponectin is being considered as one of the main adipocytokines. Adiponectin is distinguished not only as the most abundant product of fat but also as one of the major adipokines involved in regulating various mechanisms in the human body [38]. Adipose tissue also has a significant role in the control of vascular tone and blood pressure, as evidenced by the strong correlation observed between obesity, hypertension, and other cardiovascular illnesses. It is known that perivascular adipose tissue changes in its secretory profile, releasing more vasoconstricting hormones and less vasorelaxing factors [39]. Adipose tissue develops resistance to insulin and leptin and is the source of altered hormone and molecule release for adiponectin, which worsens obesity-related cardiovascular disease [40]. Additionally, the concepts of oxidative stress and endothelial dysfunction have gained interest in recent years as contributing factors in the pathogenesis of hypertension and diabetes [41].

Adiponectin is directly involved in the development of metabolic syndrome altering various clinical characteristics that make up the metabolic syndrome, such as hypertension [42]. A series of in vitro and in vivo studies suggest that adiponectin has protective actions on endothelial cells acting through the AMPK signaling pathway [43]. Moreover, plasma adiponectin levels correlated with vasodilator response to reactive hyperemia suggesting that hypoadiponectinemia is associated with impaired endothelium-dependent vasorelaxation [44].

The dynamic relationship between vasodilator and vasoconstrictor chemicals is crucial to the structural and functional integrity of the vascular wall [45]. The SHRs group, which was left untreated, had significantly higher SBP and MAP than the WKY group during the whole 28-day research. After one week of treatment with adiponectin alone or in combination with irbesartan, the SHRs' elevated blood pressure (BP) readings normalized. Furthermore, the combination of these PPAR-*γ* agonists resulted in a significant decrease in the SBP and MAP values of SHRs treated for 3 weeks individually with pioglitazone, irbesartan, and adiponectin. However, the pioglitazone treatment groups had a smaller reduction in SBP and MAP compared to the irbesartan and adiponectin combination. Increased nitric oxide (NO) generation may be responsible for adiponectin's hypotensive action [12], as NO is an endothelium-derived molecule that modulates MAP and vascular tone [46]. The vasoconstrictive effect of NO is counteracted by Ang-II, which is stimulated by the release of renin from kidney JG cells. By influencing NO production [47], this interaction with the RAAS affects blood pressure regulation. Previous findings in STZ-induced diabetic rats [48] and fructose-overloaded SD rats [46, 49] are consistent with the hypotensive effect of pioglitazone therapy. The capacity of TZDs to disrupt the RAAS has an additional profound effect on these pathways [50]. Therefore, pioglitazone's (10 mg/kg/day) antihypertensive effect is associated with an increase in plasma adiponectin and, by extension, NO generation in blood vessels, and so it possesses vasodilatory action in SHRs. Irbesartan at 30 mg/kg/day was the most effective dose for blocking RAAS, and its PPAR-*γ* agonistic function also contributed to lowering blood pressure. However, when irbesartan was used to increase PPAR-*γ* agonistic activity, combining it with adiponectin therapy resulted in an even greater drop in blood pressure in the SHRs.

Overactivity of the sympathetic nervous system (SNS) increases heart rate and peripheral vascular resistance, all of which contribute to the development of hypertension over time. [47]. Our research showed that adiponectin considerably lowered heart rate in the treated groups compared to the untreated controls. This conclusion may be attributable to adiponectin's ability to dampen the response of the sympathetic nervous system. Sympathetic nerve activity, blood pressure, and heart rate are all lowered after central delivery of adiponectin [51], which is also found in the cerebrospinal fluid [20]. Although there was no statistically significant difference in HR between the irbesartan and pioglitazone treatment groups, HR was significantly decreased in the adiponectin plus partial and full PPAR-*γ* agonist treatment groups compared to the adiponectin treatment group alone, indicating that adiponectin is an important factor in HR monitoring.

### 4.3. Arterial Stiffness

Arterial stiffness is directly proportional to impaired endothelial function, while pulse wave velocity (PWV) is deemed as its biomarker [16, 27] and vascular disease. Hence, persistent increase in B. P triggers arterial wall hypertrophy. The vascular beds of diabetic animals have been found to have impeded endothelium-dependent vasodilation [52]. In this study, the PWV of SHRs was substantially greater than that of the WKY control group, showing that the extensibility of the blood arteries in SHRs had notably reduced, which ultimately leads to increased arterial stiffness. Besides this, adiponectin's vascular protective properties are mediated through its pleiotropic activities on a variety of targets, including endothelial cells, NO concentration, and ROS [53]; therefore, its plasma concentration could be considered as a surrogate biomarker for determining its antioxidant properties. Additionally, the unbalanced level of reactive oxygen species (ROS), which includes the generation of free radicals with their reactive metabolites and antioxidants, causes damage to important biomolecules and cells of the organism [54], whereas oxidative stress (OS) and ROS exacerbate in hypertensive states [55]. Therefore, in SHRs, OS appears to be an important factor for hypertension development, whereas the reduction of OS concentration is a leading cause of PPAR-*γ* activation [56]. The production of ROS is also responsible for reducing the bioavailability of NO due to the uncoupling of eNOS [57], thus suppressing NO production and exacerbating the deterioration of eNOS activity [58]. In this study, we observed an imbalance between antioxidants and oxidative stress in terms of reduced plasma values for nitric oxide (NO) and confirmed with decrease in total antioxidant capacity (TAOC), which indicates a suppressed antioxidant defense system with free radicals production in SHRs. Previous studies also mentioned the significant role of PI3K (phosphoinositide3-kinase) in adiponectin-induced endothelial NO production in endothelial cells via AMPK activation [59, 60]; therefore, a key downstream molecule for the synthesis of adiponectin is AMPK [47]. We found that treatment with exogenous adiponectin decreased MAP and B. P values, as well as the PWV of SHRs (as explained above), indicating its potent antioxidant activity mediated by blocking its endothelial-derived NO synthase activity. Similarly, NO and TAOC plasma levels were notably elevated, indicating improved arterial stiffness in addition to reduced oxidative stress in SHRs. The study done by Agata et al. observed that ARBs, besides their antihypertensive property, act as partial PPAR-*γ* agonists and thwarts the progression of arterial stiffness [61] indirectly with an increase in plasma adiponectin and endothelial NO synthesis [62]. Moreover, TZDs also depend upon adiponectin concentration for PPAR-*γ*-mediated enhancement of endothelial function with an increase in NO bioavailability and OS reduction in diabetic states by activating signaling cascades, such as AMPK–eNOS and cAMP–PKA components, as a PPAR-*γ* agonist attenuates endothelial function [63]. Therefore, the use of pioglitazone and exogenous adiponectin together in therapy (full PPAR-*γ*) resulted to a larger decrease in PWV with an improvement in OS condition in contrast to the other sets of treatment that were tested in the study.

### 4.4. Systemic Hemodynamics

Previous reports have proven that adrenergic and Ang II-induced vasoconstriction in the rat systemic and renal vasculature is modulated by an interaction between AT1 and alpha-1-adrenoceptors [64]. Moreover, it has been proven that RAAS and SNS interrelate with each other to carry out their regulatory functions [65]. The endothelium affects the tone of the underlying VSMCs by producing vasodilator mediators [66].

SNS and RAAS contribute to the development of hypertension in diabetic rats. These systems do not function independently but mutually interact with each other in performing their regulatory functions [19]. Angiotensin II is importantly involved in contributing to the development of hypertension and the associated pathophysiology of vascular function [18]. Previous reports have proven that an interactive relationship exists between AT1 and *α*1-adrenoceptors in modulating adrenergically and Ang-II-induced vasoconstriction in the rat systemic and renal vasculature [19, 64]. Secondly, PPAR-*γ* interacts or a cross-talk relationship exists between PPAR-*γ*, alpha-adrenoceptor subtypes (*α*1A-, *α*1B-, and *α*1D), and Ang II receptors in the renal and systemic vasculature of experimental rats after adiponectin increasing plasma factors [25, 31].

One of the vital observations in the present study was the systemic vasculature's blunted vasopressor responses to adrenergic agonists including NA, PE, ME, and Ang-II in SHRs. The vascular contractility in our observations was pronounced in SHRs, which could be due to increased OS with poor endothelial activity. It was discernible that SHRs demonstrated a significant spike in presser responses to systemically administered a subset of alpha-adrenergic agonists including NA, PE, and ME. Interestingly, postsynaptic a1-adrenoceptors govern the aforementioned responses. However, treatments on a daily basis of oral pioglitazone administration (10 mg/kg), irbesartan (30 mg/kg) for 3 weeks, and one-week adiponectin to SHRs caused a reduction in systemic vascular responses to alpha-adrenergic agonists. Since both PPAR-*γ* agonists are responsible for increasing plasma adiponectin concentration, therefore, the blunted vasopressor effect of adiponectin on SNS caused a decrease in B. P and vascular tone of blood vessels, with an improvement in the SHRs' endothelial function directly. These reactions govern by diminishing the vasopressor responses to Ang-II and exogenously giving alpha-adrenergic agonists with an upsurge in plasma NO levels (as discussed above). The hypotensive and vasculoprotective effects of adiponectin result from its blunted action to heightened responsiveness to alpha-adrenergic agonists and Ang-I in SHRs with attenuation in endothelial function. It is noteworthy that Ang-II and its endothelial signaling pathways are crucial for controlling B.P. Our findings showed that irbesartan treatment attenuated vasopressor response to NA, PE, and ME, whereas interactions between RAAS and SNS have already been established [18], and this can be confirmed from the study done by Raasch et al., wherein the systemic vascular responses to NA were diminished after pretreatment with losartan [67]. However, Ang-II suggests facilitating sympathetic neurotransmission at various sites such as CNS and presynaptic sympathetic nerve terminals [68]. Additionally, there are findings that in hyperinsulinemia scenarios, pioglitazone enhances adrenergic neurons' control over the blood vessels [69, 70]. Furthermore, an enormous part is played by TZDs' capacity to interfere with RAAS [71] in mediating the vascular responsiveness to these adrenergic agonists. Prior investigations also showed that TZDs reduced the amount of Ang-I and II produced by human subcutaneous adipose tissue [72]. In contrast, it has been seen in another study that complete PPAR-agonists suppress angiotensin AT1 receptor mRNA expression in VSMCs [73]. Along with its insulin-sensitizing action, pioglitazone also exhibits a hypotensive effect due to its attenuated action to increase Ang-II responsiveness in a diabetic state by improving endothelial function [74]. Therefore, as per our study observations, pioglitazone, as a full PPAR-*γ* agonist, is also responsible for antihypertensive and vasculo-protective effects in SHRs.

Based on the above explanation, we may deduce that full and partial PPAR-*γ* agonists (pioglitazone and irbesartan), respectively, possess a broad spectrum of association with RAAS, whereas the precise extent of engagement could differ throughout systemic vasculature of SHRs in mediating NO and endothelial function while regulating blood pressure. Interestingly, alpha-adrenergic agonists were likewise attenuated by the vasopressor responses in the adiponectin-treated groups, though to a lesser extent than when it was combined with pioglitazone. However, in its cotreatment with irbesartan, presser responses to blunt Ang-II were more pronounced. The altered vascular activity and, consequently, the muted response to exogenously delivered Ang-II that are seen in the current investigation may be attributed to the increased Ang-II level. Therefore, it may be theoretically feasible to postulate that an interactive/cross-talk communication subsets between PPAR-*γ* and alpha-adrenergic agonists in the presence of adiponectin receptors in the systemic vasculature of the vasculature of genetic model of hypertension.

## 5. Conclusions

In summary, this study highlighted the following:

1. Primarily, PPAR-*γ* plays a substantial role in regulating systemic haemodynamics in SHRs. Adiponectin, as a therapeutic agent, attenuated blood pressure and systemic vascular reactivity through eNOS stimulation, therefore, improving systemic hemodynamics and ameliorating oxidative stress, which underlies its vasculoprotective role in maintaining normal systemic physiology in SHRs
2. Exogenously administered adiponectin with PPAR-*γ* agonists caused blunted systemic vascular responsiveness to *α*1-adrenergic agonists and Ang-II, which signifies a composite interaction/cross-talk between PPAR-*γ*, adiponectin receptors, and adrenoceptor subtypes, Ang-II, in systemic vasculature of SHRs
3. Adiponectin cotreatment significantly blunted vasopressor responses largely with an enhancement of oxidative state and atrial stiffness, demonstrating the amount of synergism between adiponectin and pioglitazone due to the full PPAR-*γ* agonistic effect of pioglitazone

## Figures and Tables

**Table 1:** Metabolic parameters of experimental groups treated with -*γ* agonists alone and in combination with adiponectin.

Parameters	Groups	Days of observation			
		Day 0	Day 8	Day 21	Day 28
Body Weight (g)	WKY + VEH	245 ± 5	250 ± 7	275 ± 4^∗^	289 ± 8^∗^
	SHR + VEH	242 ± 3	248 ± 6	267 ± 8^∗^	284 ± 9^∗^
	SHR + Irb	248 ± 4	252 ± 3	265 ± 7^∗^	271 ± 12^∗^
	SHR + Pio	242 ± 3	249 ± 3	263 ± 8^∗^	279 ± 8^∗^*δ*
	SHR + Adp	244 ± 2	250 ± 4	263 ± 14^∗^	247 ± 4^*δ*#ж^
	SHR + Irb + Adp	249 ± 7	251 ± 4	258 ± 5^∗^	251 ± 3^*δ*^
	SHR + Pio + Adp	244 ± 5	253 ± 6	274 ± 7^∗^^*δ*^	254 ± 9^*δζ*^

Water intake (ml/d)	WKY + VEH	43 ± 1	44 ± 2	45 ± 3	44 ± 2
	SHR + VEH	32 ± 2^^^	34 ± 2^^^	34 ± 3^^^	37 ± 4^^^
	SHR + Irb	31 ± 1	34 ± 2^∗^	35 ± 2^∗^*δ*	36 ± 2^∗*δ*#^
	SHR + Pio	30 ± 2	35 ± 3^∗^	36 ± 3^∗^	37 ± 3^∗^^ж^
	SHR + Adp	32 ± 3	33 ± 2^ж^	39 ± 1^*δ*∗^^ж^	28 ± 3^*δ*∗^
	SHR + Irb + Adp	32 ± 2	37 ± 2^∗^*δ*	36 ± 1^∗^	29 ± 1^∗*δ*^
	SHR + Pio + Adp	31 ± 2	36 ± 3^∗^	37 ± 2^∗^^*δ*^	28 ± 2^∗*δ*^

UFR (mL/min/100 g)	WKY + VEH	3.84 ± 0.44	3.63 ± 0.21	3.92 ± 0.21	3.95 ± 0.21
	SHR + VEH	3.10 ± 0.05^^^	2.98 ± 0.09^^^	2.94 ± 0.54^^^	2.93 ± 0.43^^^
	SHR + Irb	3.07 ± 0.04	3.09 ± 0.25	3.15 ± 0.28	3.19 ± 0.84^*δ*#^
	SHR + Pio	3.11 ± 0.05	3.14 ± 0.04	3.16 ± 0.04	3.25 ± 0.87^*δж*^
	SHR + Adp	3.09 ± 0.04	3.10 ± 0.02	3.10 ± 0.07	4.13 ± 0.25^*δ*^
	SHR + Irb + Adp	3.08 ± 0.03	3.09 ± 0.26	3.17 ± 0.27	4.16 ± 0.31^*δΦ*^
	SHR + Pio + Adp	3.11 ± 0.06	3.14 ± 0.04	3.17 ± 0.02	4.17 ± 0.05^*δζ*^

Notes: the values are presented as mean ± S.E.M. (*n* = 6) in each group and were analyzed by repeated measure one-way ANOVA followed by the Bonferroni *post hoc* test. Values with *P* < 0.05 were statistically significant during and at the end of treatment. ^^^A significant difference (*P* < 0.05) of SHR control in comparison to WKY control during and at the end of treatment. ^∗^A significant difference (*P* < 0.05) in comparison to day 0 of the respective group during and at end of treatment. ^*δ*^A significant difference (*P* < 0.05) of Irb, Pio, Adp, Irb + Adp, and Pio + Adp groups as compare to SHR + VEH during and at the end of treatment. ^#^A significant difference (*P* < 0.05) between Irb and Adp groups during and at the end of the treatment period. ^ж^A significant difference (*P* < 0.05) between the Pio and Adp groups during and at the end of the treatment period. ^*Φ*^A significant difference (*P* < 0.05) of the Adp group in comparison to the Irb + Adp group at days 21 and 28 of treatment. ^*ζ*^A significant difference (*P* < 0.05) of the Adp group in comparison to the Pio + Adp group at days 21 and 28 of treatment.

**Table 2:** Systemic hemodynamics parameters of experimental groups treated with -*γ* agonists alone and in combination with adiponectin.

Parameters	Groups	Days of observation			
		Day 0	Day 8	Day 21	Day 28
Systolic blood pressure (mmHg)	WKY + VEH	118 ± 5	117 ± 2	117 ± 2	120 ± 3
	SHR + VEH	159 ± 4^^^	164 ± 6^^^	162 ± 3^^^	157 ± 8^^^
	SHR + Irb	164 ± 5	150 ± 4^∗^^*δ*^	144 ± 5^∗^^*δ*^	137 ± 2^∗*δ*#^
	SHR + Pio	163 ± 2	160 ± 3	151 ± 3^∗^^*δ*^	144 ± 3^∗*δж*^
	SHR + Adp	162 ± 4	165 ± 4	160±5	129 ± 3^∗*δ*^
	SHR + Irb + Adp	166 ± 7	149 ± 5^∗^^*δ*^	145 ± 6^∗^^*δ*^	115 ± 2^∗*δΦ*^
	SHR + Pio + Adp	167 ± 2	163 ± 5	153 ± 4^∗^	135 ± 3^∗*δζ*^

Diastolic blood pressure (mmHg)	WKY + VEH	79 ± 3	84 ± 4	80 ± 3	86 ± 5
	SHR + VEH	119 ± 6^^^	117 ± 7^^^	108 ± 7^^^	120 ± 6^^^
	SHR + Irb	112 ± 8	107 ± 5	98 ± 4^∗^^*δ*^	86 ± 3^∗*δ*^
	SHR + Pio	114 ± 6	115 ± 7	113 ± 5	114 ± 7
	SHR + Adp	108 ± 9	111 ± 6	106 ± 3	95 ± 3^*δж*^
	SHR + Irb + Adp	114 ± 5	110 ± 5	98 ± 5^*δ*^	81 ± 4^∗*δ*^
	SHR + Pio + Adp	116 ± 6	102 ± 7	112 ± 7	91 ± 5

Mean arterial pressure (mmHg)	WKY + VEH	92 ± 8	97 ± 4	92 ± 4	97 ± 5
	SHR + VEH	132 ± 5^^^	133 ± 4^^^	126 ± 5^^^	132 ± 6^^^
	SHR + Irb	129 ± 6	121 ± 5^∗^^*δ*^	113 ± 3^∗^^*δ*#^	103 ± 2^∗*δ*#^
	SHR + Pio	130 ± 4	130 ± 7	126 ± 4^∗^	123 ± 5^∗*δж*^
	SHR + Adp	126 ± 6	129 ± 5	124 ± 5	106 ± 2^∗*δ*^
	SHR + Irb + Adp	130 ± 5	123 ± 5^∗^^*δ*^	114 ± 5^∗^^*δ*^	98 ± 3^∗*δΦ*^
	SHR + Pio + Adp	133 ± 6	122 ± 5	126 ± 7	120 ± 4^∗*δζ*^

Heart rate (BPM)	WKY + VEH	312 ± 10	310 ± 4	309 ± 11	303 ± 8
	SHR + VEH	386 ± 9^^^	390 ± 10^^^	392 ± 14^^^	389 ± 11^^^
	SHR + Irb	388 ± 10	385 ± 7	382 ± 5	360 ± 4^∗*δ*#^
	SHR + Pio	389 ± 10	394 ± 7	385 ± 10	372 ± 4^∗*δж*^
	SHR + Adp	395 ± 7	390 ± 9	394 ± 8^*δ*^	345 ± 11^∗*δ*^
	SHR + Irb + Adp	390 ± 8	391 ± 6	355 ± 11^*δ*^	341 ± 4^∗*δΦ*^
	SHR + Pio + Adp	388 ± 7	395 ± 7	362 ± 8^∗^^*δ*^	337 ± 8^∗*δζ*^

Notes: the values are presented as mean ± S.E.M. (*n* = 6) in each group and were analyzed by repeated measure one-way ANOVA followed by the Bonferroni *post hoc* test. Values with *P* < 0.05 were considered statistically significant. ^^^A significant difference (*P* < 0.05) of SHR control in comparison to WKY control during and at the end of treatment. ^∗^A significant difference (*P* < 0.05) in comparison to day 0 of the respective group during and at the end of treatment. ^*δ*^A significant difference (*P* < 0.05) of Irb, Pio, Adp, Irb + Adp, and Pio + Adp groups as compare to SHR + VEH during and at the end of treatment. ^#^A significant difference (*P* < 0.05) between Irb and Adp groups during and at the end of treatment. ^ж^A significant difference (*P* < 0.05) between Pio and Adp groups during and at the end of treatment. ^*Φ*^A significant difference (*P* < 0.05) of Adp groups in comparison to Irb + Adp group at days 21 and 28 of treatment. ^*ζ*^A significant difference (*P* < 0.05) of Adp groups in comparison to Pio + Adp group at days 21 and 28 of treatment.

**Table 3:** Vasopressor response to graded doses of noradrenaline, phenylephrine, methoxamine, and angiotensin II of WKY, SHR control, and SHR-treated groups on day 28.

Agonist	Dose	WKY + VEH	SHR + VEH	SHR + Irb	SHR + Pio	SHR + Adp	SHR + Irb + Adp	SHR + Pio + Adp
Noradrenaline	200 ng	20 ± 2.8	45 ± 3.2^∗^	37 ± 2.8^∗^^#*τ*^	30 ± 3.5^∗^^#^	33 ± 2.8^∗^^#^	25 ± 2.8^∗^^#*ρ*^	28 ± 2.6^#*φ*^
	400 ng	30 ± 3.3	56 ± 4.1^∗^	45 ± 3.5^∗^^#^	40 ± 3.4^∗^^#^	37 ± 3.5^∗^^#^	35 ± 3.6^#^	38 ± 3.2^∗^^#^
	800 ng	45 ± 3.9	86 ± 4.7^∗^	77 ± 4.1^∗^^#^	76 ± 3.6^∗^^#*ξ*^	61 ± 4.1^∗^^#^	60 ± 2.7^∗^^#^	50 ± 4.1^#^

Phenylephrine	2 *μ*g	30 ± 2.7	56 ± 3.2^∗^	50 ± 3.8^∗^^#*τ*^	46 ± 5.2∗^#^	38 ± 4.9∗^#^	34 ± 3.8^#^	35 ± 3.5^#^
	4 *μ*g	40 ± 3.5	70 ± 4.8^∗^	63 ± 4.5^∗^^*τ*^	56 ± 4.5^∗^^#^	50 ± 4.1^∗^^#^	53 ± 4.6^∗^^#^	46 ± 4.5^#^
	8 *μ*ng	56 ± 4.1	94 ± 5.1^∗^	85 ± 5.6^∗^^#*τ*^	77 ± 4.7^∗^^#*ξ*^	64 ± 5.2^∗^^#^	60 ± 4.7^∗^^#^	65 ± 5.2^∗^^#^

Methoxamine	2 *μ*g	10 ± 2.8	22 ± 3.2^∗^	14 ± 2.8^∗^^#^	16 ± 3.5^∗^^#^	17 ± 2.8^∗^^#^	16 ± 2.8^∗^^#^	14 ± 2.6^#^
	4 *μ*g	15 ± 3.3	35 ± 4.1^∗^	24 ± 3.5^#^	22 ± 3.4^∗^^#^	21 ± 3.5^∗^^#^	20 ± 3.6^∗^^#^	18 ± 3.2^#^
	8 *μ*ng	23 ± 3.9	47 ± 4.7^∗^	44 ± 4.1^∗^^*τ*^	32 ± 3.6^∗^^# *ξ*^	26 ± 4.1^#^	24 ± 2.7^#^	22 ± 4.1^#^

Angiotensin II	5 ng	18 ± 2.8	38 ± 3.2^∗^	21 ± 2.8^#*τ*^	34 ± 3.5^∗^	33 ± 2.8^∗^	20 ± 2.8^#*ρ*^	31 ± 2.6^∗^^#^
	10 ng	36 ± 3.3	58 ± 4.1^∗^	25 ± 3.5^∗^^#*τ*^	55 ± 3.4^∗^	54 ± 3.5^∗^	23 ± 3.6^∗^^# *ρ*^	52 ± 3.2^∗^^#^
	20 ng	50 ± 3.9	64 ± 4.7^∗^	30 ± 4.1^∗^^#*τ*^	61 ± 4.5^∗^	59 ± 4.1^∗^	29 ± 2.7^∗^^#*ρ*^	59 ± 4.1^∗^^#^

Notes: the values are Mean ± SEM (*n* = 6). Statistical analysis was done by two-way ANOVA followed by the Bonferroni *post hoc* test for all groups. Values with *P* < 0.05 were considered statistically significant. ^∗^*P* < 0.05 versus WKY; ^**#**^*P* < 0.05 versus SHR; ^*τ*^*P* < 0.05 between SHR + Irb and SHR + Adp; ^*ξ*^*P* < 0.05 between SHR+ Pio and SHR + Adp; ^*ρ*^*P* < 0.05 between SHR+ Adp to SHR + Irb + Adp; ^*φ*^*P* < 0.05 between SHR + Adp to SHR + Pio + Adp group.

## Data Availability

The analyzed data with their values have been incorporated in the tables and figures of the manuscript.
